# NF‐κB1, NF‐κB2 and c‐Rel differentially regulate susceptibility to colitis‐associated adenoma development in C57BL/6 mice

**DOI:** 10.1002/path.4527

**Published:** 2015-04-21

**Authors:** Michael D Burkitt, Abdalla F Hanedi, Carrie A Duckworth, Jonathan M Williams, Joseph M Tang, Lorraine A O'Reilly, Tracy L Putoczki, Steve Gerondakis, Rod Dimaline, Jorge H Caamano, D Mark Pritchard

**Affiliations:** ^1^ Department of Gastroenterology, Institute of Translational MedicineUniversity of LiverpoolUK; ^2^ Faculty of MedicineUniversity of TripoliTripoliLibya; ^3^ The Walter and Eliza Hall Institute of Medical ResearchMelbourneAustralia; ^4^ Department of Medical BiologyThe University of MelbourneAustralia; ^5^ Australian Centre for Blood DiseasesMonash University Central Clinical SchoolMelbourneAustralia; ^6^ Department of Cellular and Molecular Physiology, Institute of Translational MedicineUniversity of LiverpoolUK; ^7^ IBR‐MRC Centre for Immune Regulation, College of Medicine and Dental SciencesUniversity of BirminghamUK

**Keywords:** NF‐κB, dextran sulphate sodium, azoxymethane, colitis, p68 c‐Rel, colorectal cancer

## Abstract

NF‐κB signalling is an important factor in the development of inflammation‐associated cancers. Mouse models of Helicobacter‐induced gastric cancer and colitis‐associated colorectal cancer have demonstrated that classical NF‐κB signalling is an important regulator of these processes. In the stomach, it has also been demonstrated that signalling involving specific NF‐κB proteins, including NF‐κB1/p50, NF‐κB2/p52, and c‐Rel, differentially regulate the development of gastric pre‐neoplasia. To investigate the effect of NF‐κB subunit loss on colitis‐associated carcinogenesis, we administered azoxymethane followed by pulsed dextran sodium sulphate to C57BL/6, Nfkb1^−/−^, Nfkb2^−/−^, and c‐Rel^−/−^mice. Animals lacking the c‐Rel subunit were more susceptible to colitis‐associated cancer than wild‐type mice, developing 3.5 times more colonic polyps per animal than wild‐type mice. Nfkb2^−/−^ mice were resistant to colitis‐associated cancer, developing fewer polyps per colon than wild‐type mice (median 1 compared to 4). To investigate the mechanisms underlying these trends, azoxymethane and dextran sodium sulphate were administered separately to mice of each genotype. Nfkb2^−/−^ mice developed fewer clinical signs of colitis and exhibited less severe colitis and an attenuated cytokine response compared with all other groups following DSS administration. Azoxymethane administration did not fully suppress colonic epithelial mitosis in c‐Rel^−/−^ mice and less colonic epithelial apoptosis was also observed in this genotype compared to wild‐type counterparts. These observations demonstrate different functions of specific NF‐κB subunits in this model of colitis‐associated carcinogenesis. NF‐κB2/p52 is necessary for the development of colitis, whilst c‐Rel‐mediated signalling regulates colonic epithelial cell turnover following DNA damage. © 2015 The Authors. The Journal of Pathology published by John Wiley & Sons Ltd on behalf of Pathological Society of Great Britain and Ireland.

## Introduction

Chronic idiopathic inflammatory bowel diseases, including Crohn's colitis and ulcerative colitis, increase an individual's risk of developing colorectal cancer in proportion to the extent and duration of the underlying inflammatory bowel disease 1, 2, 3, 4. This suggests that chronic colonic inflammation is pivotal to the development of colon cancer, but the molecular mechanisms that influence an individual's risk of developing colitis‐associated colon cancer have not been fully established. The classical pathway of NF‐κB signalling has, however, been shown to influence both the severity of inflammation 5 and colonic carcinogenesis in animal models 6.

The NF‐κB family of proteins comprises five members: NF‐κB1 and NF‐κB2, RelA (p65), RelB, and c‐Rel. These proteins bind DNA as homo‐ and hetero‐dimers that influence gene expression upon interaction with the basal transcriptional machinery. Engagement of tumour necrosis factor receptor (TNFR)‐1 activates the classical NF‐κB pathway leading to the translocation of NF‐κB1 p50/RelA or p50/c‐Rel heterodimers into the nucleus. These induce the expression of genes that encode pro‐inflammatory cytokines and anti‐apoptotic proteins. In contrast, ligation of other TNF family receptors such as CD40 or lymphotoxin β receptor induces both the classical and the alternative/non‐canonical NF‐κB signalling pathways. The latter results in the processing of the NF‐κB2 p100 precursor and nuclear translocation of NF‐κB2 p52/RelB heterodimers, which control the expression of chemokines and cell adhesion molecules involved in lymphoid tissue development and B‐cell maturation 7, 8, 9.

Many studies investigating the expression and function of NF‐κB subunits have highlighted the importance of classical NF‐κB signalling, particularly involving RelA, in epithelial pathology. There is increasing evidence that other NF‐κB subunits also influence epithelial pathology. For example, c‐Rel, which is expressed in the epidermis and hair follicles of embryonic mice, functions in conjunction with RelA to promote basal cell proliferation and hair follicle formation 10. We have also recently demonstrated that RELA, RELB, NF‐κB1 p100/p50, and NF‐κB2 p100/p52 are expressed in the gastric mucosa of wild‐type mice and that specific members of the NF‐κB family of proteins differentially regulate the development of *Helicobacter felis*‐associated carcinogenesis. Mice lacking the NF‐κB1 p105/p50 subunit (*Nfkb1^−/−^*) develop gastric atrophy of greater severity than wild‐type mice following *H. felis* infection, whilst mice lacking the p100/p52 subunit (*Nfkb2^−/−^*) were protected from developing gastric mucosal lesions even after prolonged exposure to *H. felis* 11.

Evidence from transgenic mouse models has suggested that classical NF‐κB signalling exerts a complex influence over the development of colitis 5 and colon cancer 6. Conditional deletion of *Ikkb* under the control of the *villin* promoter is an established model of intestinal epithelial cell‐specific abrogation of classical NF‐κB signalling. When exposed to dextran sulphate sodium (DSS), these mice exhibited an impaired healing response; however, when crossed with interleukin 10‐deficient mice in standard animal house conditions, no difference in the severity of colitis was observed between mice with abrogated NF‐κB signalling and littermate controls 5. Mice lacking *Ikkb* in intestinal epithelial cells have also been reported to have an increased susceptibility to developing colitis‐associated dysplasia 6. When administered a single dose of azoxymethane (AOM) followed by pulsed administration of DSS, more adenomata developed in the colonic mucosa of *Ikkb*‐deficient mice than in their wild‐type counterparts. Furthermore, these mice showed elevated apoptotic indices following AOM/DSS administration. This suggests that altered susceptibility to programmed cell death influences the initiation of colonic carcinogenesis in this model. Despite these findings, the role(s) of specific NF‐κB subunits in inflammation‐associated colon cancer development remains unknown. To address this, we have studied the effects of DSS/AOM‐induced colitis‐associated carcinogenesis in mice carrying germline deletions of the *Nfkb1*, *Nfkb2* or *c‐Rel* genes and have also investigated the impact of these deletions on DSS‐induced colitis and colonic responses to DNA damage.

## Materials and methods

### Mice

Transgenic animals were maintained on a C57BL/6 genetic background. Wild‐type controls were sourced from either Charles River (Margate, UK) or the Walter and Eliza Hall Institute (WEHI). Procedures were performed with ethical approval under UK Home Office licences or following the guidelines of the WEHI Medical Research Animals Ethics Committee. Homozygote colonies of *Nfkb1^−/−^* 12, *Nfkb2^−/−^* 13, *c‐Rel^−/−^* 14, and *Tpl2^−/−^* 15 mice were maintained in conventional animal house conditions. Procedures were performed on young adult (10–12 weeks) male mice at the University of Liverpool and on young adult female mice at the WEHI. All control data presented are from age‐ and sex‐matched mice maintained in the same vivarium as relevant test animals. Control mice were either bred and maintained on‐site (WEHI) or acclimatized to local animal house conditions prior to treatment (Liverpool).

### Induction of azoxymethane and dextran sodium sulphate (DSS)‐induced adenomas

Studies were performed at both the University of Liverpool and the WEHI, Melbourne. At each institution, age‐ and sex‐matched mice were used as controls, but some differences in protocol were maintained due to differences in response to DSS in local vivarium conditions. At the University of Liverpool, groups of ten male mice were administered 12.5 mg/kg azoxymethane (Sigma‐Aldrich, Gillingham, UK) by i.p. injection 5 days before supplementation of drinking water with 0.5% DSS w/v (MW: 36–50 000; MP Biomedicals, Loughborough, UK) offered *ad libitum* for 5 days, followed by 16 days' recovery. Second and third cycles of 0.75% w/v DSS for 5 days were commenced at days 21 and 42. Animals were culled on day 63. At the WEHI, female mice were treated and the dose of DSS was increased to 2% w/v. Animals were culled at 80 days.

### Induction of colitis by DSS

Drinking water was supplemented with 2% DSS for 5 days and animals were euthanized at day 6. Clinical disease activity indices were recorded daily 16. Morphological assessment was performed by a board‐accredited veterinary pathologist (JW). Quantitative histology was completed using an established inflammation scoring system 16; all scorers were blinded to genotype and treatment whilst scoring.

### DNA damage by azoxymethane or γ‐irradiation and crypt survival assay

Mice were administered 10 mg/kg azoxymethane via a single i.p. injection. Cell turnover was quantified on a cell positional basis using morphological criteria 17. Whole‐body γ‐irradiation was administered to mice via a closed‐source ^137^Cs irradiator. Surviving crypts were scored per colonic circumference; a surviving crypt was defined as containing ten or more adjacent healthy‐looking epithelial cells and a lumen 18.

### Immunohistochemistry and real‐time PCR (RT‐PCR)

Tissue sections were immunolabelled for Ki67 (M724901; DAKO, Ely, UK), cleaved caspase 3 (AF835; R&D Systems, Abingdon, UK) or pH2AX (#9718; Cell Signaling, Beverley, MA, USA). Ki67‐ and caspase 3‐positive cells within adenomas were quantified in three high‐power fields (×40 objective) per mouse and the mean was calculated. Cell positional scoring was performed as previously described 19. For RT‐PCR, epithelial cell‐enriched samples were prepared using a modified Weiser technique 20. All assays were performed in a Roche LightCycler 480 machine. An apoptosis‐regulating gene PCR array (SABiosciences, Crawley, UK; see Supplementary Table 1 for gene list) was performed on pooled samples. Other qPCR assays were performed using individual samples, primers and probes used are identified in supplementary table 2. Samples were normalized to *Gapdh* and are represented as linearized fold changes.

### Statistics

Normally distributed data were analysed by one‐ and two‐way ANOVAs with Dunnett's *post‐hoc* analysis. Non‐parametric data were assessed by Kruskal‐Wallis one‐way ANOVA with Dunn's *post‐hoc* analysis. Cell positional statistics were analysed by median testing as previously described 17. Statistical tests achieving *p* < 0.05 were considered significant.

## Results

### Deletion of specific NF‐κB subunits alters susceptibility towards AOM/DSS‐induced colonic carcinogenesis

**Figure 1:**
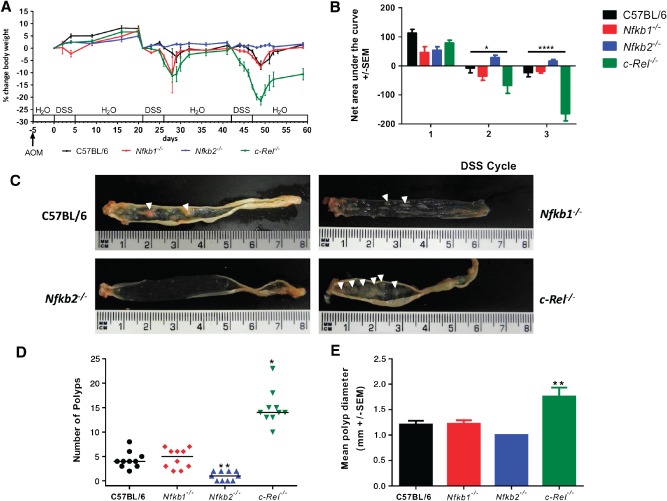
AOM and pulsed low‐dose DSS in C57BL/6, Nfkb1^−/−^, Nfkb2^−/−^, and c‐Rel^−/−^ mice. (A) Percentage change of body weight during AOM and DSS exposure. (B) Area under the curve analysis for panel A. Data separated into first, second, and third DSS cycles. Differences tested by two‐way ANOVA and Dunnett's post‐hoc test. *p < 0.05, ****p < 0.0001 for c‐Rel^−/−^ relative to wild type (WT). (C) Gross colonic pathology from mice of each genotype. Polypoid colonic lesions are marked with an arrowhead. (D) Number of polyps per mouse; horizontal line at median. Differences tested by Kruskal–Wallis one‐way ANOVA and Dunn's multiple comparison tests. *p < 0.05, **p < 0.01 versus WT. (E) Mean polyp diameter per mouse. Differences tested by one‐way ANOVA and Dunnett's post‐hoc test. **p < 0.01 versus WT. Ten mice per group.

Groups of ten male mice were administered 12.5 mg/kg azoxymethane followed by three pulses of DSS. Wild‐type mice exhibited a modest clinical response to this regime. Their weight increased consistently following the first cycle of 0.5% w/v DSS [8% (± SEM 0.95)]; hence the dose was increased to 0.75% w/v DSS for subsequent cycles. The second cycle of DSS caused a peak weight loss of 3.6% (±2.25%); the third cycle of DSS caused further weight loss (7.0% ± 1.85%) (Figure 1A). *Nfkb1^−/−^* mice developed a similar pattern of clinical signs and area under the curve (AUC) analysis demonstrated no significant differences in the severity scores between wild‐type mice and *Nfkb1^−/−^* mice (Figure 1B). *Nfkb2^−/−^* mice gained weight following each pulse of DSS (5.0% ± 0.62%, 2.3% ± 0.59%, and 1.7% ± 0.57%) (Figures 1A and 1B). In contrast, *c‐Rel^−/−^* mice exhibited a greater weight loss than wild‐type mice in response to both the second (11.3% ± 6.9%, *p* < 0.05) and the third (19.5% ± 3.3%, *p* < 0.0001) cycle of DSS (Figures 1A and 1B).

**Figure 2:**
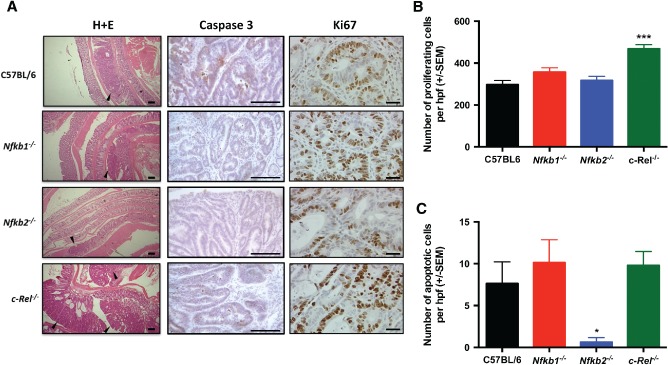
Histology induced by AOM/DSS in C57BL/6, Nfkb1^−/−^, Nfkb2^−/−^, and c‐Rel^−/−^ mice. (A) H&E‐, cleaved caspase 3‐, and Ki67‐stained sections from mice following DSS/AOM treatment. Arrowheads highlight adenomas. **(**B) Mean number of Ki67‐positive cells per hpf within adenomas. (C) Mean number of cleaved caspase 3‐positive cells per hpf within adenomas. Significant differences tested by one‐way ANOVA and Dunnett's test for multiple comparisons. *p < 0.05, ****p < 0.0001 versus WT. Ten mice per group.

Wild‐type and *Nfkb1^−/−^* mice developed a median of four colonic polyps per animal (PPA). *Nfkb2^−/−^* mice developed fewer polyps (median 1 PPA, *p* < 0.01), whilst *c‐Rel^−/−^* mice showed an increased number (median 14 PPA, *p* < 0.05). The polyps in *c‐Rel^−/−^* mice were larger than those seen in wild‐type mice (mean diameter 1.8 mm versus 1.2 mm in wild‐type, *p* < 0.01, Figures 1C–1E). Colonic polyps were adenomas by histological examination (Figure 2A). Adenomas from wild‐type mice had a mean Ki67^+^ proliferating cell index of 297 ± 20.4 cells per high power field (hpf); adenomas examined from *Nfkb1^−/−^* and *Nfkb2^−/−^* mice had similar proliferative indices. In contrast, adenomas in *c‐Rel^−/−^* mice had higher proliferation indices, with a mean of 468 ± 20.5 proliferating cells per hpf (Figures 2A, right‐hand column, and 2B, *p* < 0.0001). Apoptotic cells within adenomas were identified by cleaved caspase 3 immunostaining (Figures 2A and 2C). Adenomas from wild‐type mice had an apoptotic index of 7.6 ± 2.6 cells per hpf, which was similar in *Nfkb1^−/−^* and *c‐Rel^−/−^* mice. However, the adenomas that arose in *Nfkb2^−/−^* mice exhibited a lower apoptotic index (0.6 ± 1.8 cells per hpf, *p* < 0.05).

P105/NF‐κB1, the precursor of the p50/NF‐κB1 transcription factor, binds to and stabilizes TPL2, a serine/threonine MAP3 kinase responsible for ERK activation during inflammatory responses 15. The absence of detectable TPL2 in *Nfkb1^−/−^* cells prompted us to determine whether a loss of this kinase activity contributed to the phenotype seen in the *Nfkb1^−/−^* mice. No significant difference in visible polyps, colon length, polyp number or diameter was observed in AOM/DSS‐treated *Tpl2^−/−^* mice (Supplementary Figure 1). These findings indicate that neither the direct transcriptional regulatory function of NF‐κB1 nor its impact on TPL2‐dependent ERK/MAPK signalling substantially influences AOM/DSS‐induced colitis‐associated adenoma formation.

Collectively, these observations demonstrate that deletion of c‐Rel and Nfkb2 has inverse effects on colonic adenoma development following DSS/AOM. Deletion of Nfkb2 led to a reduced tumour burden, whilst deletion of c‐Rel led to substantially increased adenoma formation.

### DSS‐induced inflammation is enhanced by signalling through NF‐κB2

**Figure 3:**
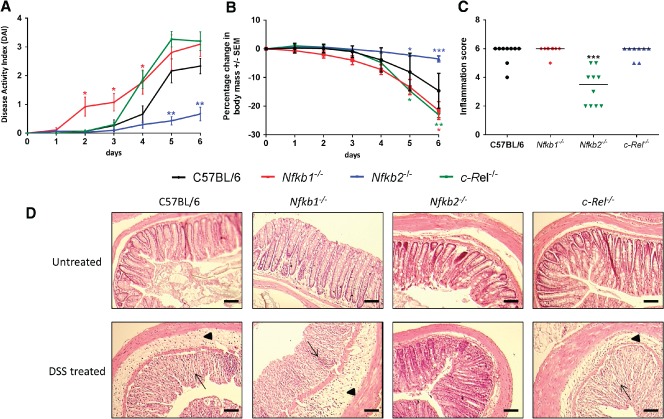
Impact of acute DSS administration on C57BL/6, Nfkb1^−/−^, Nfkb2^−/−^, and c‐Rel^−/−^ mice. (A) Clinical disease activity index plotted daily during DSS administration and recovery. Differences tested by Kruskal–Wallis one‐way ANOVA and Dunn's multiple comparison test at each time point. *p < 0.05, **p < 0.01 relative to WT. (B) Percentage change in body weight each day during DSS administration (mean and SEM). (C) Histological inflammation score per mouse; horizontal line at median. Differences tested by one‐way ANOVA and Dunnett's multiple comparison test. *p < 0.05, **p < 0.01, ***p < 0.001 versus WT. (D) Representative H&E‐stained sections of distal colon from mice following 2% DSS treatment (7–10 mice per group). Arrowheads highlight submucosal oedema; arrows highlight almost complete loss of colonic epithelium.

The AOM/DSS model involves the administration of two stimuli; we examined the effects that loss of specific NF‐κB proteins had on each of these stimuli alone. Groups of seven to ten male mice were exposed to 2% w/v DSS for 5 days and culled on day 6. This dose schedule has previously been shown to induce moderately severe colitis in C57BL/6 mice in our animal house environment. *Nfkb1^−/−^* mice developed severe diarrhoea, rectal bleeding, and weight loss earlier than wild‐type counterparts, and showed higher disease activity scores 16 than wild‐type mice at days 2–4 (*p* < 0.05). *Nfkb2^−/−^* mice exhibited less severe disease activity indices than their wild‐type counterparts on days 5 and 6 (*p* < 0.05) (Figure 3A). By the end of the experimental procedure, all mice in the wild‐type, *Nfkb1^−/−^*, and *c‐Rel^−/−^* groups had developed clinical colitis with weight loss and diarrhoea; several mice also developed haematochezia. Two of ten *Nfkb2^−/−^* mice developed diarrhoea; none exhibited haematochezia. AUC analysis of the clinical colitis index plot (*p* < 0.05) and weight monitoring confirmed a less severe clinical course for *Nfkb2^−/−^* mice compared with wild‐type mice (Figures 3A and 3B). Colonic mucosa from wild‐type mice treated with DSS exhibited almost complete effacement of normal architecture, with loss of surface and crypt epithelium; a marked, predominantly neutrophilic, inflammatory cell infiltrate; and submucosal oedema (Figure 3D). Similar changes were observed in colonic tissues from DSS‐treated *Nfkb1^−/−^* and *c‐Rel^−/−^* mice. The crypts and surface epithelia of DSS‐treated *Nfkb2^−/−^* mice were more frequently intact. To quantify these effects, a visual analogue histological inflammation score was used 16: wild‐type, *Nfkb1^−/−^*, and *c‐Rel^−/−^* mice all had median inflammation scores of 6, whilst the median inflammation score for *Nfkb2^−/−^* mice was lower at 3.5 (*p* < 0.005, Figure 3C).

**Figure 4:**
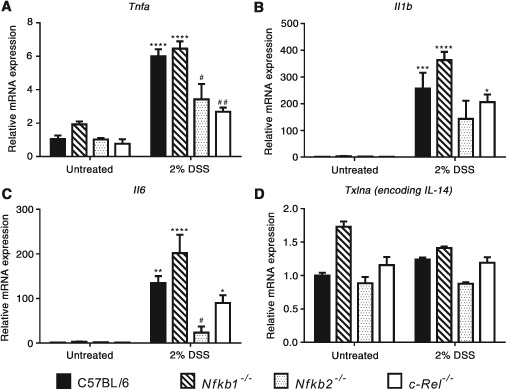
(A–D) Relative expression of the indicated cytokines in untreated and 2% DSS‐treated mice determined by real‐time PCR. Comparisons. * denotes significant difference between untreated and DSS‐treated mice of the same genotype. ^#^ denotes significant difference between DSS‐treated WT and DSS‐treated transgenic mice tested by two‐way ANOVA and Dunnett's multiple comparison test. One symbol = p < 0.05; two symbols = p < 0.01; three symbols = p < 0.001; four symbols = p < 0.0001 (four mice per group).

To determine the mechanisms underlying the differences in inflammatory response to DSS, epithelial cell‐enriched samples were prepared for RNA extraction from mice exposed to DSS. Expression of a panel of cytokines was assayed by real‐time PCR. Wild‐type mice demonstrated a 6‐fold increase in *Tnfa*, a > 200‐fold increase in *Il1b*, and a > 100‐fold increase in *Il6* transcript abundance following DSS treatment (Figure 4). *Txlna* mRNA was not altered. A similar pattern of cytokine production was observed in mutant mice, but of differing magnitude that was strain‐dependent. *Nfkb1^−/−^* and wild‐type mice had similar levels of transcript abundance. Similarly, *c‐Rel^−/−^* mice showed an increased abundance of each cytokine following DSS treatment, but only the differences in the abundance of *Il1b* and *Il6* reached statistical significance in this genotype. *Tnfa* transcripts were significantly lower in DSS‐treated *c‐Rel^−/−^* mice than similarly treated wild‐type mice; however, this did not correlate with any reduction in the clinical or histopathological severity of colitis. In *Nfkb2^−/−^* mice, there was a generalized attenuation in cytokine response following DSS administration. There was an increase in *Tnf, Il1b*, and *Il6* in response to DSS, but the differences between untreated *Nfkb2^−/−^* mice and DSS‐treated *Nfkb2^−/−^* mice did not reach statistical significance (Figure 4). Overall, these observations demonstrate that signalling through NF‐κB2/p52 is necessary for DSS to induce colitis at these doses, whereas NF‐κB1/p50 and c‐Rel appear to have little role in the regulation of DSS‐induced colitis.

### Specific NF‐κB subunits differentially regulate epithelial responses to DNA‐damaging agents

**Figure 5:**
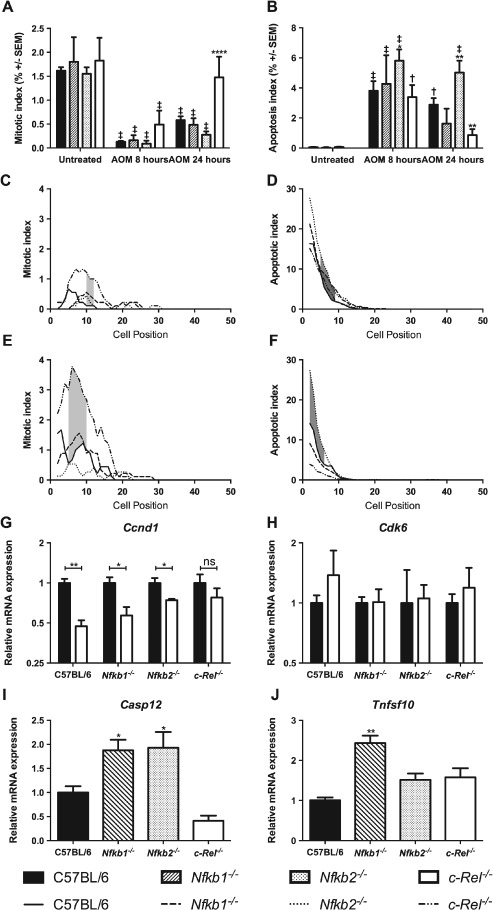
Effect of administration of 10 mg/kg AOM on cell turnover in the distal colon 8 and 24 h after administration in C57BL/6, Nfkb1^−/−^, Nfkb2^−/−^, and c‐Rel^−/−^ mice. (A) Mean percentage of cells morphologically mitotic in untreated mice and mice 8 or 24 h following AOM treatment. (B) Mean percentage of cells morphologically apoptotic in untreated mice and mice 8 or 24 h following AOM treatment. Differences were tested by two‐way ANOVA and Dunnett's test for multiple comparisons. * denotes significant difference between AOM‐treated WT and AOM‐treated transgenic mice at the same time point. *p < 0.05, **p < 0.01, ****p < 0.0001. ^†^ and ^‡^ denote significant difference between untreated and AOM‐treated mice of the same genotype. ^†^ p < 0.01, ^‡^ p < 0.0001. All analyses performed on groups of six mice. (C–F) Cell positional plots of mitotic cells (C, E) or apoptotic cells (D, F) 8 h (C, D) or 24 h after treatment with AOM (E, F). Shaded areas identify cell positions where a significant difference in mitotic index was detected between WT and c‐Rel^−/−^ mice (C, E), or in apoptotic index WT and Nfkb2^−/−^ mice (D, F) by modified median test, p < 0.05. (G, H) Relative expression of the indicated mRNAs in colonic mucosa of untreated mice (black bars) and mice 8 h after AOM administration (white bars). Differences tested by two‐way ANOVA and Dunnett's test for multiple comparisons; *p < 0.005, **p < 0.001 versus untreated mice of the same genotype. ns = not significant. (I, J) Relative expression of the indicated mRNAs in colonic mucosa 8 h after AOM administration. Significant differences in linearized expression values tested in n = 4 mice by one‐way ANOVA and Dunnett's test for multiple comparisons. *p < 0.005, **p < 0.001 versus WT.

To determine whether mice lacking specific NF‐κB subunits had different colonic epithelial responses to DNA damage, 10 mg/kg AOM was administered to groups of at least six mice per genotype and colonic tissue samples were isolated 8 or 24 h later. Cells undergoing mitosis or apoptosis were quantified by morphological criteria and cell positional scoring of H&E‐stained sections. This method of quantification was validated in this setting by cell positional scoring of sections immunostained for Ki67 and cleaved caspase 3. These alternative methods of quantification validated the initial morphological scores, which were adopted elsewhere (Supplementary Figure 2). AOM suppressed colonic epithelial cell mitosis in wild‐type, *Nfkb1^−/−^*, and *Nfkb2^−/−^* mice at both time points (Figure 5A). Cell proliferation in *c‐Rel^−/−^* mice recovered more rapidly than in wild‐type mice, with similar amounts of mitosis to the untreated state being observed 24 h after AOM treatment (Figure 5A, *p* < 0.0001). Persistent mitoses were identified in *c‐Rel^−/−^* mice between cell positions 9 and 11 at 8 h (Figure 5C, shaded region) and between cell positions 5 and 10 at 24 h after AOM administration (Figure 5E, shaded region).

As previously described 21, administration of AOM induced increased apoptosis in wild‐type mice (Figure 5B, 77‐fold increase at 8 h, p < 0.0001). Similar apoptosis scores were observed 8 h after AOM treatment in mice lacking specific NF‐κB subunits. Twenty‐four hours after AOM treatment, there were signs that the apoptotic response to AOM differed amongst the mutant animals. Nfkb2^−/−^ mice exhibited more colonic epithelial cell apoptosis than wild‐type mice (1.75‐fold increase, p < 0.01, Figure 5B). Less colonic epithelial apoptosis was observed in c‐Rel^−/−^ mice than in wild‐type mice at this time point (3.3‐fold less, p < 0.01, Figure 5B). Colonic epithelial cell apoptosis was more frequent in AOM‐treated Nfkb2^−/−^ mice than in wild‐type mice between cell positions 4 and 9 at 8 h (Figure 5D) and between positions 1 and 5 at 24 h (Figure 5F). This suggests that Nfkb2^−/−^ mice have a more robust apoptotic response to AOM administration than wild‐type mice, whilst c‐Rel^−/−^ mice undergo less cell death than wild‐type mice following the same stimulus.

To confirm these observations, and to examine underlying mechanisms, real‐time PCR was performed on epithelial cell‐enriched samples to determine the expression of genes involved in cell cycle progression and apoptosis. Since the phenotypes observed in these mice, particularly the differential apoptotic responses, were most pronounced morphologically at 24 h, we planned to identify underlying molecular mechanisms at the earlier 8 h time point when precipitating cellular events may be occurring. Messenger RNA was extracted from colonic tissue samples from at least four mice per genotype following treatment with AOM. Transcript abundance of Ccnd1 (encoding cyclin D1) was suppressed by DNA damage in wild‐type, Nfkb1^−/−^, and Nfkb2^−/−^ mice, but not in c‐Rel^−/−^ mice following AOM administration (Figure 5G). The abundance of Cdk6 mRNA was unaltered in these conditions, suggesting that either this target is not involved in cell cycle arrest induced by AOM or its effects are not modulated by transcriptional regulation in this setting (Figure 5H). Apoptotic pathways affected by AOM administration were identified by RT‐PCR array. Since at 8 h, Nfkb2^−/−^ mice exhibited the most marked increase in apoptotic response to AOM compared with wild‐type mice, we chose to compare these two groups. Ten apoptosis‐regulating targets demonstrated > 2‐fold changes; however, once targets with poor melt curve characteristics, or low abundance, had been excluded, the most significant changes were observed in Casp12 and Tnsf10. Casp12 encodes an inflammatory caspase that has been shown to be involved in the pathogenesis of both experimental colitis and colitis‐associated cancer models 18, whilst Tnfs10 encodes the TNF family ligand TRAIL, which signals through the classical NF‐κB pathway. This protein has been shown to regulate apoptosis both through a caspase‐8‐dependent mechanism 22 and in inflammatory settings 23. Real‐time PCR was performed to investigate the expression of these transcripts in other genotypes. Casp12 was up‐regulated in both Nfkb2^−/−^ and Nfkb1^−/−^ mice following AOM treatment (Figure 5I). A 2.4‐fold increase in Tnfs10 mRNA abundance was observed in Nfkb1^−/−^ mice following AOM administration, but the increased transcript abundance of Tnfs10 in Nfkb2^−/−^ mice compared with wild‐type did not reach statistical significance (Figure 5J). Other regulators of apoptosis under the transcriptional regulation of NF‐κB pathway signalling including xIAP, cIAP1, and cIAP2 were not significantly altered following AOM administration. These data suggest that c‐Rel and Nfkb2/p52 regulate responses to DNA damage in the colon. Loss of c‐Rel activity promotes cell survival following DNA damage. In contrast, absence of Nfkb2/p52 favours cell death.

### An absence of c‐Rel confers protection from γ‐irradiation‐induced colonic crypt destruction

**Figure 6:**
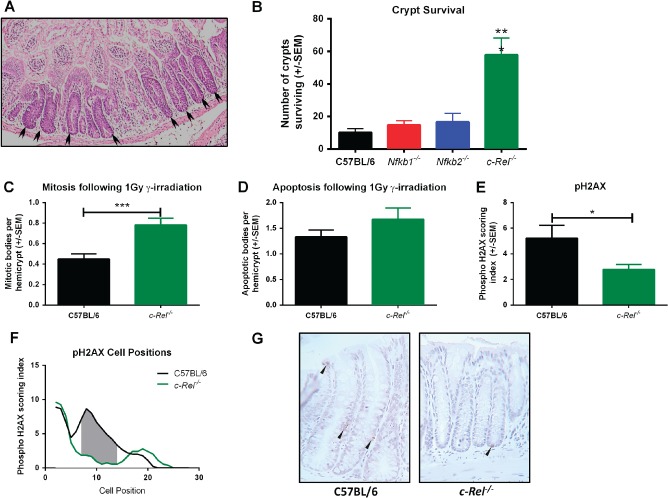
Crypt survival following γ‐irradiation in the colons of C57BL/6, Nfkb1^−/−^, Nfkb2^−/−^, and c‐Rel^−/−^ mice. (A) H&E‐stained sections of c‐Rel^−/−^colon 96 h following 12 Gy γ‐irradiation. Arrowheads highlight regenerating crypts. (B) Mean number of surviving crypts per circumference of colon 96 h following 12 Gy γ‐irradiation. Differences tested by one‐way ANOVA and Dunnett's multiple comparison test. ***p < 0.001 versus WT (six mice per group). (C–E) Mean percentages of colonocytes from C57BL/6 or c‐Rel^−/−^ mice with morphologically mitotic (C), morphologically apoptotic (D), or expressing pH2AX (E) 4.5 h following 1 Gy γ‐irradiation (two‐tailed Student's t‐test *p < 0.05, ***p < 0.001). (F) Cell positional plot of pH2AX‐expressing cells 4.5 h following 1 Gy γ‐irradiation. Shaded area marks cell positions where a significant difference in pH2AX staining index was detected by modified median test, p < 0.05. (G) Representative photomicrographs of pH2AX staining in colonic mucosa following 1 Gy γ‐irradiation.

Previous studies have demonstrated that when DNA damage‐induced apoptosis and suppression of mitosis are impaired, colonic crypts may inappropriately survive that stimulus. This effect can be modelled by the assessment of crypt survival and regeneration 96 h following sub‐lethal γ‐irradiation 18. Therefore a dose of 12 Gy whole‐body γ‐irradiation was administered as a single fraction to groups of six male mice of each genotype. Animals were maintained for 96 h prior to histological examination of the colon. Regenerating or surviving crypts were identifiable by reported morphological criteria (Figure 6A) 18. c‐Rel^−/−^ mice had enhanced crypt survival compared with wild‐type counterparts (5.7‐fold increase, p < 0.001, Figure 6B); other transgenic groups were not significantly different from wild‐type mice. To investigate the response to γ‐irradiation further, c‐Rel^−/−^ and wild‐type mice were exposed to 1 Gy whole‐body γ‐irradiation and culled 4.5 h later. In these animals, persistent mitosis was observed in the c‐Rel^−/−^ group together with an attenuated DNA damage response, as evidenced by a reduction in serine‐139 phosphorylated histone H2AX between cell positions 7 and 14 (Figures 6C–6G). This is concordant with our findings following AOM administration and offers one mechanism by which c‐Rel^−/−^ mice may retain DNA‐damaged cells in the epithelium and hence become more prone to AOM/DSS‐induced colonic tumourigenesis.

## Discussion

Previous studies have identified NF‐κB signalling pathways as regulators of intestinal inflammation and carcinogenesis. Greten *et al* demonstrated that classical NF‐κB signalling influences the development of colonic cancers in experimental colitis‐associated cancer models, and that both epithelial cells and myeloid lineages are involved in these processes 6. The data presented here demonstrate that different NF‐κB subunits have distinct regulatory functions that influence colonic epithelial responses to both DNA damage and inflammation, and highlight the particular importance that the c‐Rel subunit plays in preventing colonic pathology.

There appears to be a hierarchy of effects associated with each of the NF‐κB subunits examined in these experiments. An absence of *Nfkb1* had the least impact on the phenotype observed in wild‐type mice, with similar numbers and sizes of tumours being observed following AOM/DSS administration. In keeping with this finding, the effect that a lack of NF‐κB1 had on colonic responses to either DSS colitis or DNA damage in isolation was minimal. These observations provide an intriguing contrast to previous studies in *Nfkb1^−/−^* mice. Work from our laboratory demonstrated spontaneous gastric mucosal inflammation and a more severe phenotype in response to chronic *Helicobacter felis* infection, whilst Wang *et al* have previously shown that *Nfkb1^−/−^* mice are more susceptible to radiation‐induced small intestinal apoptosis than wild‐type mice 24. This reflects the already established evidence of a complex relationship between NF‐κB1/P50 mediated signalling and inflammatory responses in other settings 25. We wanted to identify whether the known pathways that involve NF‐κB1 signalling were influencing colonic carcinogenesis in tandem – hence our investigation of the *Tpl2^−/−^* mouse. However, our findings suggest that neither classical NF‐κB signalling utilizing NF‐κB1 nor Tpl2‐mediated activation of ERK/MAPK influences the AOM/DSS model. The response of *Nfkb1^−/−^* mice to inflammatory stimuli therefore remains difficult to predict, possibly reflecting pathway redundancy between NF‐κB signalling proteins, which may allow the phenotype induced by *Nfkb1* deletion to be rescued in some tissue contexts but not others.

Signalling mediated by c‐Rel appears to have an important role in the pathogenesis of DSS/AOM‐induced colonic tumours. Mice lacking this subunit developed more severe colonic adenomatous disease, with more abundant and larger neoplastic lesions than wild‐type mice. In our model of DSS‐induced colitis, *c‐Rel^−/−^* mice developed inflammation and cytokine responses similar to those of wild‐type mice, suggesting that a change in inflammatory phenotype is not the predominant cause of altered response to DSS/AOM. Intriguingly, polymorphisms in *REL*, the human homologue of c‐Rel, have been identified as an IBD susceptibility locus, with the minor allele conferring an increased risk of both Crohn's disease 26 and ulcerative colitis 27. Considering our observation that the response to DSS was unaltered in *c‐Rel^−/−^* mice, the mechanism responsible for the increased IBD susceptibility of certain *REL* alleles remains unclear.

DNA damage responses in *c‐Rel^−/−^* mice were different from other groups of mice. A higher percentage of proliferating cells were observed in the colonic epithelium of this mutant strain following low‐dose γ‐irradiation and AOM treatment; this correlated with an attenuated DNA damage response. These changes in cellular response serve to enhance cell survival following DNA damage, an event that was confirmed by the observation of greater colonic crypt survival in *c‐Rel^−/−^* than in wild‐type mice following γ‐irradiation. The observation that an absence of c‐Rel did not inhibit epithelial cell turnover is intriguing as it runs counter to observations in lymphoid cells, where c‐Rel appears to be critical for mitogen‐induced cell division and survival 28.

Current genome‐wide association studies investigating IBD cohorts have not addressed whether risk alleles for IBD also alter an individual's susceptibility to colitis‐associated cancer, and at present, there are no published data on whether known polymorphisms at the *REL* locus influence the risk of either colitis‐associated cancer or sporadic colorectal cancer in humans. Given our observations and the association of *REL* polymorphisms with primary sclerosing cholangitis 29, a colitis‐associated cancer‐predisposing condition, this appears to be a pertinent question to address in the future. In contrast to deletion of *c‐Rel*, *Nfkb2^−/−^* mice exhibited an attenuated response to DSS/AOM. Compared with wild‐type, *Nfkb2^−/−^* mice developed fewer, smaller tumours. The response of *Nfkb2^−/−^* mice to DSS colitis was also strikingly less severe than that of mice of other genotypes.

Overall, these findings suggest that signalling mediated by c‐Rel is required to select appropriate damaged cycling cells within the colonic mucosa to undergo senescence or apoptosis. Loss of this function potentially creates a permissive environment for the survival of cells harbouring mutations which may become the foci for initiation of colonic carcinogenesis. In contrast, NF‐κB2‐mediated signalling appears to be critical for the onset of DSS‐induced colitis. Hence *Nfkb2^−/−^* animals subjected to DSS and AOM were protected from developing colitis‐associated colonic neoplasia primarily by an attenuated inflammatory response. These studies highlight the complex interactions between individual NF‐κB subunits in regulating colonic susceptibility to inflammation and neoplasia, and in particular highlight the critical role for signalling via c‐Rel in the regulation of colonic cell turnover.

## Author contribution statement

MDB contributed to data acquisition, analysis, and interpretation, and drafted the manuscript. AFH contributed to data acquisition and analysis. CAD, JMW, JMT, LAO'R, and TLP contributed to data acquisition and analysis and drafting of the manuscript. SG contributed to data analysis and drafting of the manuscript. RD contributed to project concept and design. JHC contributed to project concept, provided original transgenic mouse colonies, and contributed to manuscript drafting. DMP contributed to data analysis and interpretation and was project lead for concept, design, and supervision of the project.

**SUPPORTING INFORMATION ON THE INTERNET**

The following supporting information may be found in the online version of this article:

**Figure S1.** Impact of AOM and pulsed low‐dose DSS on C57BL/6, *Nfkb1^−/−^*, and *Tpl2^−/−^* mice.

**Figure S2.** Quantification of Ki67‐ and caspase 3‐stained colonic epithelial cells in untreated mice and mice treated with a single dose of 10 mg of AOM 24 h prior to necropsy.

**Table S1.** Genes assayed by apoptosis‐regulating gene real‐time PCR array.

**Table S2.** Primers and probes used for real‐time PCR assays.

## Supporting information


**FigureS1.** Impact of AOM and pulsed low‐dose DSS on C57BL/6, Nfkb1^−/−^ and Tpl2^−/−^ mice. A: Representative images of gross colonic pathology from mice of each genotype showing polyploid colonic lesions, examples are marked with an arrowhead. B: Plot of colon length per mouse. C: Dot‐plot demonstrating number of polyps per mouse; horizontal line at median. D: Plot of mean polyp diameter per mouse. E: Representative photomicrographs of H&E‐stained sections of distal colon from mice following AOM and pulsed low‐dose DSS. Statistics tested by 1‐way ANOVA. 5 female mice per group.

**FigureS2.** Quantification of Ki67‐ and caspase 3‐stained colonic epithelial cells in untreated mice and mice treated with a single dose of 10 mg of AOM 24 h prior to necropsy. A and B show percentage of epithelial cells marked by IHC. C and D plot the percentage of stained cells on a cell positional basis. *p < 0.05; **p < 0.01 by 2‐way ANOVA.

**TableS1.** Genes assayed by apoptosis‐regulating gene real‐time PCR array

**TableS2.** Primers and probes used for real‐time PCR assays
