# Supplementary figures and images for: NF‐κB1, NF‐κB2 and c‐Rel differentially regulate susceptibility to colitis‐associated adenoma development in C57BL/6 mice
Source: J Pathol. 2015 Apr 21;236(3):326–36. doi: 10.1002/path.4527 (PMC4737252; doi:10.1002/path.4527)

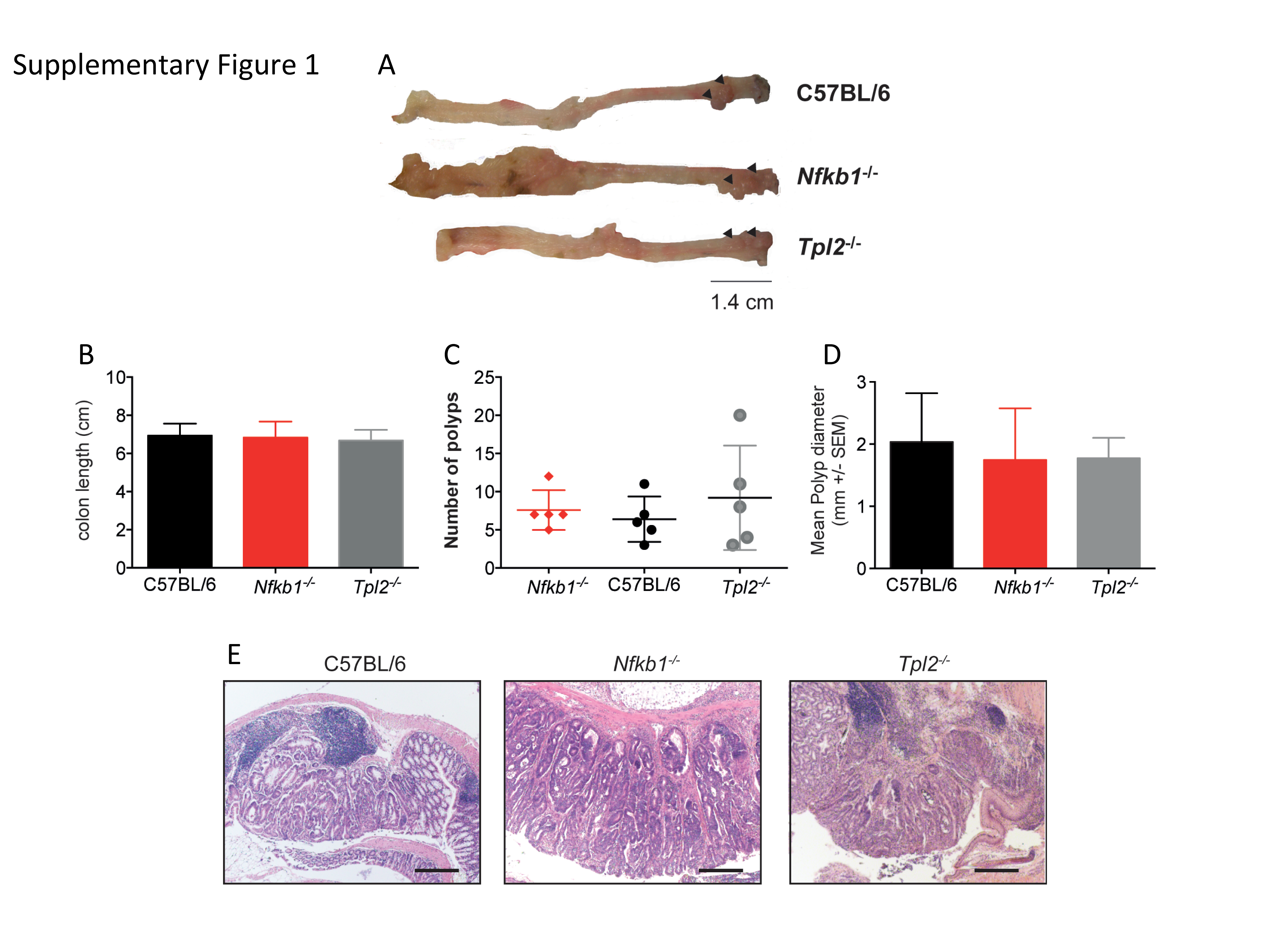

Supplement: Supplementary file 1 — FigureS1. Impact of AOM and pulsed low‐dose DSS on C57BL/6, Nfkb1−/− and Tpl2−/− mice. A: Representative images of gross colonic pathology from mice of each genotype showing polyploid colonic lesions, examples are marked with an arrowhead. B: Plot of colon length per mouse. C: Dot‐plot demonstrating number of polyps per mouse; horizontal line at median. D: Plot of mean polyp diameter per mouse. E: Representative photomicrographs of H&E‐stained sections of distal colon from mice following AOM and pulsed low‐dose DSS. Statistics tested by 1‐way ANOVA. 5 female mice per group. [file PATH-236-326-s001.tif]
