# Supplementary material for: NF‐κB1, NF‐κB2 and c‐Rel differentially regulate susceptibility to colitis‐associated adenoma development in C57BL/6 mice
Source: J Pathol. 2015 Apr 21;236(3):326–36. doi: 10.1002/path.4527 (PMC4737252; doi:10.1002/path.4527)
Supplement: Supplementary file 3 — TableS1. Genes assayed by apoptosis‐regulating gene real‐time PCR array [file PATH-236-326-s003.docx]

**Supplementary Table 1.** Genes assayed by apoptosis-regulating gene real-time PCR array

| **Symbol** | **Description** |  | **Symbol** | **Description** |
| --- | --- | --- | --- | --- |
| Akt1 | Thymoma viral proto-oncogene 1 |  | Dad1 | Defender against cell death 1 |
| Apaf1 | Apoptotic peptidase activating factor 1 |  | Dapk1 | Death associated protein kinase 1 |
| Api5 | Apoptosis inhibitor 5 |  | Dffa | DNA fragmentation factor, alpha subunit |
| Atf5 | Activating transcription factor 5 |  | Dffb | DNA fragmentation factor, beta subunit |
| Bad | BCL2-associated agonist of cell death |  | Tsc22d3 | TSC22 domain family, member 3 |
| Bag1 | Bcl2-associated athanogene 1 |  | Fadd | Fas (TNFRSF6)-associated via death domain |
| Bag3 | Bcl2-associated athanogene 3 |  | Fas | Fas (TNF receptor superfamily member 6) |
| Bak1 | BCL2-antagonist/killer 1 |  | Fasl | Fas ligand (TNF superfamily, member 6) |
| Bax | Bcl2-associated X protein |  | Hells | Helicase, lymphoid specific |
| Bcl10 | B-cell leukemia/lymphoma 10 |  | Il10 | Interleukin 10 |
| Bcl2 | B-cell leukemia/lymphoma 2 |  | Lhx4 | LIM homeobox protein 4 |
| Bcl2l1 | Bcl2-like 1 |  | Ltbr | Lymphotoxin B receptor |
| Bcl2l10 | Bcl2-like 10 |  | Mcl1 | Myeloid cell leukemia sequence 1 |
| Bcl2l2 | Bcl2-like 2 |  | Nfkb1 | Nuclear factor of kappa light polypeptide gene enhancer in B-cells 1, p105 |
| Bid | BH3 interacting domain death agonist |  | Nme5 | Non-metastatic cells 5, protein expressed in (nucleoside-diphosphate kinase) |
| Naip1 | NLR family, apoptosis inhibitory protein 1 |  | Nol3 | Nucleolar protein 3 (apoptosis repressor with CARD domain) |
| Naip2 | NLR family, apoptosis inhibitory protein 2 |  | Pak7 | P21 protein (Cdc42/Rac)-activated kinase 7 |
| Birc2 | Baculoviral IAP repeat-containing 2 |  | Pim2 | Proviral integration site 2 |
| Birc3 | Baculoviral IAP repeat-containing 3 |  | Polb | Polymerase (DNA directed), beta |
| Xiap | X-linked inhibitor of apoptosis |  | Prdx2 | Peroxiredoxin 2 |
| Birc5 | Baculoviral IAP repeat-containing 5 |  | Pycard | PYD and CARD domain containing |
| Bnip2 | BCL2/adenovirus E1B interacting protein 2 |  | Ripk1 | Receptor (TNFRSF)-interacting serine-threonine kinase 1 |
| Bnip3 | BCL2/adenovirus E1B interacting protein 3 |  | Rnf7 | Ring finger protein 7 |
| Bnip3l | BCL2/adenovirus E1B interacting protein 3-like |  | Sphk2 | Sphingosine kinase 2 |
| Bok | BCL2-related ovarian killer protein |  | Tnf | Tumor necrosis factor |
| Card10 | Caspase recruitment domain family, member 10 |  | Tnfrsf10b | Tumor necrosis factor receptor superfamily, member 10b |
| Nod1 | Nucleotide-binding oligomerization domain containing 1 |  | Tnfrsf11b | Tumor necrosis factor receptor superfamily, member 11b |
| Card6 | Caspase recruitment domain family, member 6 |  | Tnfrsf1a | Tumor necrosis factor receptor superfamily, member 1a |
| Casp1 | Caspase 1 |  | Cd40 | CD40 antigen |
| Casp12 | Caspase 12 |  | Tnfsf10 | Tumor necrosis factor (ligand) superfamily, member 10 |
| Casp14 | Caspase 14 |  | Tnfsf12 | Tumor necrosis factor (ligand) superfamily, member 12 |
| Casp2 | Caspase 2 |  | Cd40lg | CD40 ligand |
| Casp3 | Caspase 3 |  | Cd70 | CD70 antigen |
| Casp4 | Caspase 4, apoptosis-related cysteine peptidase |  | Traf1 | Tnf receptor-associated factor 1 |
| Casp6 | Caspase 6 |  | Traf2 | Tnf receptor-associated factor 2 |
| Casp7 | Caspase 7 |  | Traf3 | Tnf receptor-associated factor 3 |
| Casp8 | Caspase 8 |  | Trp53 | Transformation related protein 53 |
| Casp9 | Caspase 9 |  | Trp53bp2 | Transformation related protein 53 binding protein 2 |
| Cflar | CASP8 and FADD-like apoptosis regulator |  | Trp53inp1 | Transformation related protein 53 inducible nuclear protein 1 |
| Cidea | Cell death-inducing DNA fragmentation factor, alpha subunit-like effector A |  | Trp63 | Transformation related protein 63 |
| Cideb | Cell death-inducing DNA fragmentation factor, alpha subunit-like effector B |  | Trp73 | Transformation related protein 73 |
| Cradd | CASP2 and RIPK1 domain containing adaptor with death domain |  | Zc3hc1 | Zinc finger, C3HC type 1 |
