# Supplementary material for: NF‐κB1, NF‐κB2 and c‐Rel differentially regulate susceptibility to colitis‐associated adenoma development in C57BL/6 mice
Source: J Pathol. 2015 Apr 21;236(3):326–36. doi: 10.1002/path.4527 (PMC4737252; doi:10.1002/path.4527)
Supplement: Supplementary file 4 — TableS2. Primers and probes used for real‐time PCR assays [file PATH-236-326-s004.docx]

**Supplementary Table 2.** Primers and probes used for real-time PCR assays

| **Target** | **Forward primer** | **Reverse primer** | **UPL probe** | **Amplicon length** |
| --- | --- | --- | --- | --- |
| *Il1b* | TTG-ACG-GAC-CCC-AAA-AGA-T | GAA-GCT-GGA-TGC-TCT-CAT-CTG | #26 | 75 |
| *Tnf* | TCT-TCT-CAT-TCC-TGC-TTG-TGG | GGT-CTG-GGC-CAT-AGA-ACT-GA | #49 | 128 |
| *Il6* | GCT-ACC-AAA-CTG-GAT-ATA-ATC-AGG-A | CCA-GGT-AGC-TAT-GGT-ACT-CCA-GAA | #6 | 78 |
| *Txlna* | GAG-AAG-CTG-GCT-GCA-CTG-T | TCA-TCT-GCT-TCT-GCG-AGT-TC | #11 | 73 |
| *Tnfsf10* | GCT-CCT-GCA-GGC-TGT-GTC | CCA-ATT-TTG-GAG-TAA-TTG-TCC-TG | #76 | 87 |
| *Casp12* | TGA-TGC-TTT-TTA-TGT-CCA-GGA-GT | TGG-ATC-TCT-TTC-ATG-TGT-CCT-C | #76 | 67 |
| *Ccnd1* | GAG-ATT-GTG-CCA-TCC-ATG-C | CTC-CTC-TTC-GCA-CTT-CTG-CT | #67 | 78 |
| *Cdk6* | GCC-CTT-ACC-TCG-GTG-GTC | ACA-GGG-GTG-GCA-TAG-CTG | #15 | 77 |
| *Gapdh* | GGG-TTC-CTA-TAA-ATA-CGG-ACT-GC | CCA-TTT-TGT-CTA-CGG-GAC-GA | #52 | 112 |
